# Prevalence, Risk Factors, and Awareness of Erectile Dysfunction in the Saudi Arabian Population

**DOI:** 10.7759/cureus.61233

**Published:** 2024-05-28

**Authors:** Basel O Hakami, Alwaleed A Alhazmi, Abdulaziz M Kariri, Faisal A Zaybi, Abdulrahman W Hadadi, Naif K Mahzara, Feras A Ageeli, Abdulrahman A Aqeel, Faisal H Mahzari, Meshari A Alzahrani

**Affiliations:** 1 Department of Urology, King Faisal Medical City for Southern Region (KFMC), Abha, SAU; 2 Department of Urology, King Fahad Central Hospital, Jazan, SAU; 3 Faculty of Medicine, Jazan University, Jazan, SAU; 4 Department of Urology, College of Medicine, Majmaah University, Al Majmaah, SAU

**Keywords:** risk factors, awareness, prevalence, psychological triggers, erectile dysfunction

## Abstract

Background: Erectile dysfunction (ED) is defined as the inability to achieve and maintain an erection powerful enough to permit pleasurable sexual activity. There are four categories for ED grades. The illness may be influenced by vascular, neurological, psychological, and hormonal factors. Anxiety about performance and relationship issues are common psychological triggers.

Aim: This study aimed to determine the prevalence, risk factors, and awareness of ED and its management in the population of Saudi Arabia.

Method: This community-based, cross-sectional study was conducted among adult Saudi males in all five regions of Saudi Arabia (Central, Eastern, Western, Southern, and Northern). A self-administered questionnaire was distributed among participants using an online survey. The questionnaire includes sociodemographic data (i.e., age, region, marital status, education), medical history, and erectile function (International Index of Erectile Function (IIEF-5)) as a diagnostic tool for ED.

Results: In total, 924 men took part. About 512 (55.4%) were aged between 18 and 25 years old, and nearly two-thirds (595, 64.4%) were single. The Internet was the most common source of ED information 495 (53.6%). Based on respondents' knowledge, the most common risk factor of ED was depression (561, 60.8%), while the most common treatment option was lifestyle modification (654, 70.8%). The prevalence of ED among adult Saudi men was 198 (21.4%). Independent risk factors for ED include having been married, being an employee, and previous operation of the perineum.

Conclusion: ED was common among the Saudi male population. ED was more prevalent among older men with associated chronic diseases and had elevated body mass index (BMI). Having been married, being an employee, and having a previous perineum operation were identified as the significant independent risk factors for ED. Longitudinal studies are needed to determine the cause and effect of the recognized risk factors for ED among men.

## Introduction

Erectile dysfunction (ED) is a prevalent condition that significantly impacts the sexual health and overall quality of life for men worldwide. Characterized by the persistent difficulty in achieving or maintaining an erection sufficient for satisfactory sexual performance, ED affects men of all ages but is particularly prevalent among older adults [1,2]. The condition is multifaceted, involving a complex interplay of vascular, neurological, psychological, and hormonal factors. Psychological triggers such as performance anxiety or relationship issues frequently contribute to its onset, while lifestyle choices and certain medications, including antidepressants and tobacco use, are known to exacerbate the problem [3,4].

ED is not merely a discomforting sexual health issue but also a potential indicator of underlying systemic health problems [5-7]. Conditions such as diabetes, hypertension, obesity, and cardiovascular diseases are commonly associated with the incidence of ED, making it a relevant marker for overall men’s health. Additionally, sociodemographic factors such as age, marital status, and socioeconomic status have been identified as influential, with varying prevalence and awareness across different cultures and regions. This variability underscores the necessity of regional studies to understand the dynamics of ED more comprehensively and to tailor public health policies and healthcare services accordingly [8,9].

The objective of this study is to explore the prevalence, risk factors, and awareness of erectile dysfunction and its management specifically in the Saudi Arabian population. This focus is pertinent given the unique sociocultural and health landscape of Saudi Arabia, which may influence both the manifestation of ED and the approaches to its management. This research aims to fill the gap in localized data, contributing to more effective, culturally sensitive health interventions that address both the physiological and psychological dimensions of ED within this demographic. Understanding these aspects can guide healthcare providers in developing targeted treatment and prevention programs, ultimately improving patient outcomes and wellbeing in the region.

## Materials and methods

Study overview

This descriptive cross-sectional study was conducted in a community setting and engaged 924 participants. These individuals were recruited through convenience and snowball sampling methods, utilizing social media and email to collect participants. The main aim of the study is to explore the prevalence and factors associated with ED among men in the community.

Ethical Considerations

The study adheres to ethical guidelines appropriate for community-based research, ensuring informed consent, confidentiality, and participants' right to withdraw at any time. Ethical approval was obtained from a suitable review board before the commencement of the study.

Study Criteria

Participants included in the study were men aged 18 years and above. The exclusion criteria were set to omit individuals below this age to maintain a focus on an adult population.

Procedure

Eligible respondents were asked to complete self-administered questionnaires to gather data relevant to the study objectives. The approach ensures privacy and comfort, encouraging more honest and accurate responses.

Tools

The study utilized digital tools for data collection, including social media platforms and email, to distribute and collect the questionnaires, facilitating a wider reach within the community.

Assessments

ED was assessed using an abridged version of the International Index of Erectile Function (IIEF-5) [8], comprising five questions related to sexual function. Each question had five response options, allowing a total possible score of 25.

Sample Size Calculation

The sample size of 924 was determined using the single proportion sample size formula: N = (Zα)² p(1-p)/d², where N = required sample size, Zα = Z-score corresponding to the desired level of confidence (e.g., 1.96 for a 95% confidence level), and p = estimated proportion or prevalence of the characteristic of interest, d = margin of error (5% in this study). This calculation assumed a 95% confidence level.

Statistical analysis

Data were analyzed using the IBM SPSS Statistics for Windows, Version 26 (Released 2019; IBM Corp., Armonk, New York, United States). Categorical variables were presented as numbers and percentages, while continuous variables were summarized using means and standard deviations. The Chi-square test was utilized to examine the relationship between the prevalence of ED and sociodemographic characteristics, as well as medical history. Significant associations were further explored using multivariate regression analysis to identify significant independent risk factors for ED, reporting odds ratios and 95% confidence intervals. A p-value of 0.05 was used to determine statistical significance.

## Results

This study enrolled 924 adult Saudi males. As given in Table 1, 512 (55.4%) were aged between 18 and 25 years. Respondents who lived in the Eastern Region constituted 335 (36.3%). Respondents who were single constituted 595 (64.4%). Concerning education, 620 (67.1%) were bachelor's degree holders and 426 (46.1%) were employed. In addition, 223 (24.1%) were classified as obese (Table 1).

**Table 1:** Sociodemographic characteristics of Saudi male participants (n = 924) BMI: Body mass index

Study data	N (%)
Age group	
18-25 years	512 (55.4%)
26-35 years	240 (26.0%)
36-45 years	81 (08.8%)
>45 years	91 (09.8%)
Region	
Central Region	92 (10.0%)
Eastern Region	335 (36.3%)
Western Region	219 (23.7%)
Southern Region	270 (29.2%)
Northern Region	08 (0.90%)
Marital status	
Single	595 (64.4%)
Married	323 (35.0%)
Divorced	04 (0.40%)
Widowed	02 (0.20%)
Educational level	
Primary school	01 (0.10%)
Intermediate school	05 (0.50%)
Secondary school	293 (31.7%)
Diploma holder	03 (0.30%)
Bachelor degree	620 (67.1%)
Postgraduate	02 (0.20%)
Professional status	
Unemployed	414 (44.8%)
Self-employed	53 (05.7%)
Employed	426 (46.1%)
Retired	31 (03.4%)
BMI level	
Underweight (<18.5 kg/m2)	54 (05.8%)
Normal (18.5–24.9 kg/m2)	370 (40.0%)
Overweight (25–29.9 kg/m2)	277 (30.0%)
Obese (≥30 kg/m2)	223 (24.1%)

The rate of current smokers was 246 (26.6%). Of them, 106 (30.3%) took at least 1 to 10 cigarettes daily. Only 13 (1.4%) were current alcohol drinkers. The prevalence of men with hypertension, diabetes, and depression was 58 (6.3%), 51 (5.5%), and 123 (13.3%), respectively. Respondents who were taking antidepressants were 37 (4%). Respondents who were diagnosed with urinary tract infection (UTI) were 58 (6.3%), while those who suffered an injury in the perineal area were 32 (3.5%). Respondents who underwent an operation on the perineum were 14 (1.5%). The proportion of participants who believed that ED can be treated was 684 (74%) (Table 2).

**Table 2:** Medical history (n = 924)

Variables	N (%)
Smoking status	
Current smoker	246 (26.6%)
Ex-smoker	104 (11.3%)
Nonsmoker	574 (62.1%)
Number of cigarette smoke per day ^(n = 350)^	
Not mentioned	70 (20.0%)
1-10	106 (30.3%)
11-40	82 (23.4%)
41-80	44 (12.6%)
>80	48 (13.7%)
Do you drink alcohol?	
Yes, I drink now	13 (01.4%)
Yes, I drank before	47 (05.1%)
No, I didn't drink	864 (93.5%)
Do you have hypertension?	
Yes	58 (06.3%)
No	866 (93.7%)
Do you have diabetes?	
Yes	51 (05.5%)
No	873 (94.5%)
Do you suffer from depression?	
Yes	123 (13.3%)
No	801 (86.7%)
Do you take medication for depression?	
Yes	37 (04.0%)
No	887 (96.0%)
Have you been diagnosed with a urinary tract infection?	
Yes	58 (06.3%)
No	866 (93.7%)
Have you ever suffered an injury in the perineal area?	
Yes	32 (03.5%)
No	892 (96.5%)
Have you had an operation on the perineum?	
Yes	14 (01.5%)
No	910 (98.5%)
Can erectile dysfunction be treated?	
Yes	684 (74.0%)
No	11 (01.2%)
I don't know	229 (24.8%)

**Figure 1:**
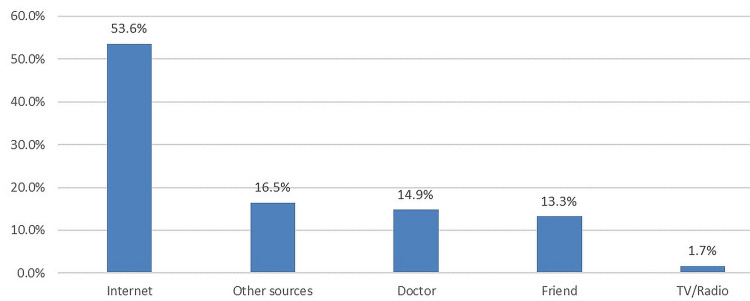
Source of erectile dysfunction information Other sources: News papers, magazines, and social media

In Figure 1, the most common source of ED information was the Internet (495, 53.6%), followed by the doctor (137, 14.9%) and a friend (123, 13.3%).

**Figure 2:**
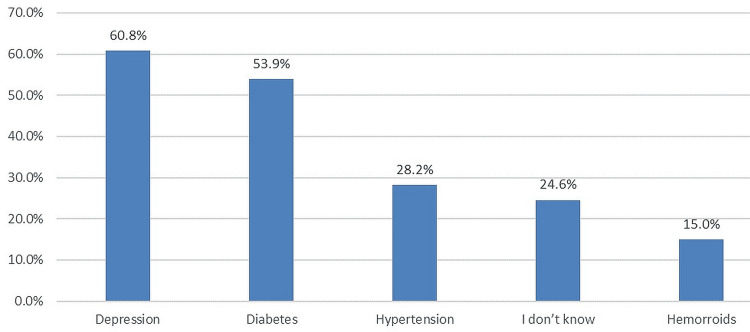
Respondents' knowledge, the most common risk factor for ED ED: Erectile dysfunction

In Figure 2, according to respondents' knowledge, the most common risk factor for ED was depression (561, 60.8%), followed by diabetes (498, 53.9%) and hypertension (260, 28.2%).

**Figure 3:**
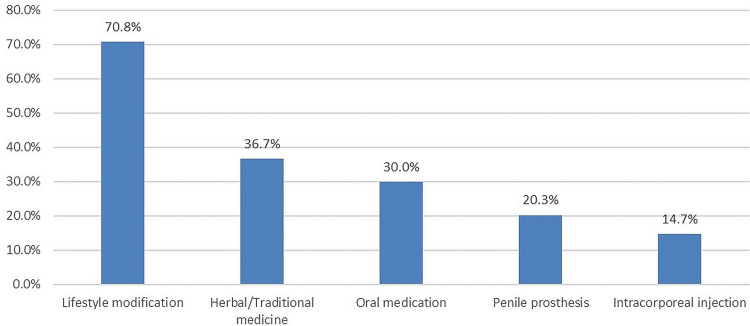
Knowledge about the most prominent treatment option for ED ED: Erectile dysfunction

In Figure 3, the most prominent treatment option for ED was lifestyle modification (654, 70.8%), followed by herbal/traditional medicine (339, 36.7%) and oral medication (277, 30%).

Table 3 shows that the total mean score of IIEF-5 was 23.5 (SD 2.78), with normal, mild, mild to moderate, moderate, and severe levels constituting 597 (64.6%), 129 (14%), 186 (20.1%), 10 (1.1%), and 2 (0.2%), respectively. Accordingly, the prevalence of ED among Saudi male respondents was 198 (21.4%), and the rest were non-ED (726, 78.6%).

**Table 3:** Prevalence of erectile dysfunction (ED) according to IIEF-5 (n = 924) IIEF-5: International Index of Erectile Function

Variables	N (%)
IIEF-5 score (mean ± SD)	23.5 ± 2.78
Severity of ED	
Normal (score 22-25)	597 (64.6%)
Mild (score 17-21)	129 (14.0%)
Mild to moderate (score 12-16)	186 (20.1%)
Moderate (score 8-11)	10 (01.1%)
Severe (score 1-7)	02 (0.20%)
Prevalence of ED	
ED (score 1-16)	198 (21.4%)
Non-ED (score 17-25)	726 (78.6%)

When measuring the relationship between ED according to the sociodemographic characteristics and previous medical history (Table 4), it was observed that the prevalence of ED was significantly more common among the older age group (p < 0.001), those who had been married (p < 0.001), employed (p < 0.001), those with elevated body mass index (BMI) (p = 0.011), current/ex-smoker (p = 0.020), hypertension (p = 0.005), diabetes (p = 0.005), previous diagnosis of UTI (p = 0.030), and previous operation on the perineum (p = 0.009) (Table 4).

**Table 4:** Relationship between erectile dysfunction among the sociodemographic characteristics of previous medical history (n = 924) BMI: Body mass index ^§^ p-value has been calculated using Chi-square test. ** Significant at p < 0.05 level

Factor	Prevalence of ED		p-value ^§^
	ED N (%) ^(n = 198)^	Non-ED N (%) ^(n = 726)^	
Age group			
≤25 years	44 (22.2%)	468 (64.5%)	<0.001 **
>25 years	154 (77.8%)	258 (35.5%)	
Marital status			
Never been married	45 (22.7%)	550 (75.8%)	<0.001 **
Been married	153 (77.3%)	176 (24.2%)	
Educational level			
Secondary or below	61 (30.8%)	241 (33.2%)	0.526
Bachelor degree	137 (69.2%)	485 (66.8%)	
Professional status			
Unemployed	40 (20.2%)	405 (55.8%)	<0.001 **
Employed	158 (79.8%)	321 (44.2%)	
BMI level			
Normal or underweight	75 (37.9%)	349 (48.1%)	0.011 **
Overweight or obese	123 (62.1%)	377 (51.9%)	
Smoking status			
Current/ex-smoker	83 (41.9%)	267 (36.8%)	0.186
Nonsmoker	115 (58.1%)	459 (63.2%)	
Do you drink alcohol?			
Current/ex-drinker	20 (10.1%)	40 (05.5%)	0.020 **
Nondrinker	178 (89.9%)	686 (94.5%)	
Do you have hypertension?			
Yes	21 (10.6%)	37 (05.1%)	0.005 **
No	177 (89.4%)	689 (94.9%)	
Do you have diabetes?			
Yes	19 (09.6%)	32 (04.4%)	0.005 **
No	179 (90.4%)	694 (95.6%)	
Do you suffer from depression?			
Yes	20 (10.1%)	103 (14.2%)	0.134
No	178 (89.9%)	623 (85.8%)	
Do you take medication for depression?			
Yes	08 (04.0%)	29 (04.0%)	0.977
No	190 (96.0%)	697 (96.0%)	
Have you been diagnosed with a urinary tract infection?			
Yes	19 (09.6%)	39 (05.4%)	0.030 **
No	179 (90.4%)	687 (94.6%)	
Have you ever suffered an injury in the perineal area?			
Yes	10 (05.1%)	22 (03.0%)	0.168
No	188 (94.9%)	704 (97.0%)	
Have you had an operation on the perineum?			
Yes	07 (03.5%)	07 (01.0%)	0.009 **
No	191 (96.5%)	719 (99.0%)	

When conducting multivariate regression estimates, it was revealed that respondents who had been married and employed and had undergone an operation on the perineum were identified as the significant independent risk factors for ED. This further suggests that compared to unmarried males, males who had been married were predicted to increase the risk of ED by at least 7.5 times higher (adjusted odds ratio (AOR) = 7.512; 95% CI = 4.465-12.634; p < 0.001). Employed participants were at increased risk of ED by at least 1.7-fold higher than those who were unemployed (AOR = 1.061-2.752; p = 0.027). Also, we observed that respondents who underwent an operation in the perineum were 3.7 times more likely to have an increased risk for ED (AOR = 3.711; 95% CI = 1.034-13.327; p = 0.044). Other variables included in the model did not show a significant effect after regression adjustments, including age, BMI level, drinking alcohol, hypertension, diabetes, and previous diagnosis of UTI (p > 0.05) (Table 5).

**Table 5:** Multivariate regression analysis to determine the significant independent risk factors associated with erectile dysfunction (n = 924) BMI: Body mass index; AOR: adjusted odds ratio; CI: confidence interval ** Significant at p < 0.05 level

Factor	AOR	95% CI	p-value
Age group			
≤25 years	Ref		
>25 years	1.246	0.708-2.191	0.446
Marital status			
Never been married	Ref		
Been married	7.512	4.465-12.634	<0.001 **
Professional status			
Unemployed	Ref		
Employed	1.709	1.061-2.752	0.027 **
BMI level			
Normal or underweight	Ref		
Overweight or obese	0.931	0.634-1.367	0.716
Do you drink alcohol?			
Current/ex-drinker	Ref		
Nondrinker	0.724	0.368-1.422	0.348
Do you have hypertension?			
Yes	1.056	0.552-2.020	0.868
No	Ref		
Do you have diabetes?			
Yes	1.205	0.607-2.395	0.594
No	Ref		
Have you been diagnosed with a urinary tract infection?			
Yes	0.897	0.454-1.772	0.753
No	Ref		
Have you had an operation on the perineum?			
Yes	3.711	1.034-13.327	0.044 **
No	Ref		

## Discussion

This study investigated the prevalence, risk factors, awareness, and management of ED among adult Saudi men. In this study, the prevalence of ED was 198 (21.4%). Regarding ED severity, we observed that 129 (14%), 186 (20.1%), 10 (1.1%), and 2 (0.2%) of the respondents were considered mild, mild to moderate, moderate, and severe levels, respectively. This is comparable to the study done in the USA [6], reporting an overall prevalence of ED of 18.4%. Similarly, Ahn et al. [8] documented a prevalence of self-reported ED at 13.4%. However, based on the IIEF-5 criteria, this has increased to 32.4%. Contradicting these reports, several studies documented a higher prevalence of ED among men, ranging from 40% to 86.1% [9-18]. The highest ED prevalence has been reported by El-Sakka and Tayeb [7], reporting a prevalence of 86.1%, with severe levels showing 49.1%. The prevalence of ED varies according to region and the questionnaire criteria being utilized. Many studies used an IIEF-5 cutoff point of <22 points [7,10,15,18], while some studies used ≤17 [8,19]. Other studies used different methods [6,20,21]. Our study used a cutoff point of ≤17 to determine ED prevalence in men. ED is a common problem of men in society, but it is unspoken. Hence, awareness campaigns are necessary to address the high prevalence of ED in adult men.

According to our results, there was a high prevalence of ED detected in older men, having been married, being employed, having an elevated BMI, being a current/ex-alcohol drinker, and having a previous operation in the perineum (all p < 0.05). However, in our multivariate regression model, having been married, being employed, and having a prior operation in the perineum are the only variables that remained significant and determined as the significant independent risk factors for ED. These findings are in agreement with the report of Zhang et al. [9]. According to their results, increasing age was found to be associated with increasing risk for ED, with smoking more than 30 cigarettes per day and obesity identified as the significant risk factor for ED based on a multivariate AOR. Similarly, Abu et al. [20] also noted that age was a risk factor for ED, with risk being significantly higher in men aged between 60 and 79 years. In another study conducted by Li et al. [22], modifiable lifestyle risk factors such as smoking, alcohol drinking, lack of physical exercise, and elevated BMI were prevalent among older men with ED. They added that men with ED were less likely to work than men without ED.

In the study, 44 young males under the age of 25 experienced ED, highlighting the importance of addressing this issue in younger populations. Common causes of ED in this age group include psychological factors such as stress and anxiety, lifestyle influences like substance use, smoking, and poor diet, as well as medical conditions such as diabetes and cardiovascular issues. Effective management strategies should involve a comprehensive approach including medical evaluation to identify and treat any underlying health conditions, lifestyle modifications like improving diet and increasing physical activity, and psychological support to address mental health issues like anxiety and depression. Additionally, pharmacological treatments such as phosphodiesterase type 5 (PDE5) inhibitors may be considered where appropriate, alongside education and open communication about sexual health to reduce stigma and improve understanding around ED.

Studies suggest that the risk of ED increases among men with associated chronic disease. For instance, Ponholzer et al. [10] documented that diabetes, hyperlipidemia, lower UTI, and psychological stress were recognized as the significant risk factors for ED. This is corroborated by the study of Khatib et al. [15], who reported that glycemic control, coronary artery disease, hypertension, neuropathy, and retinopathy were known to increase the risk for ED. Our study supported these findings, as we also detected that hypertension, diabetes, and previous history of UTI were more prevalent among men with ED. However, in our multivariate regression model, these variables did not remain significant and were not identified as independent risk factors for ED. Incidentally, according to the systematic review of Miller [11], evidence suggests that glycemic control positively correlated with ED in most studies, but smoking and hypertension were not. On the other hand, physical activity was determined as the protective factor for ED, as the study concluded.

Surprisingly, most of our subjects (684, 74%) knew that ED is treatable, and they knew that the most common risk factors for ED were depression (561, 60.8%), diabetes (498, 53.9%), and hypertension (260, 28.2%), only 227 (24.6%) were not aware of these risk factors. In Jeddah City [16], it was documented that the most common risk factor among patients with ED was the lack of exercise (82%) and smoking (56%). Other risk factors being recognized were taking regular medications (44%), diabetes (30%), a history of pelvic surgery (14%), and alcoholism (13%). 

Moreover, we noticed that our population was seen to have a lack of awareness regarding help-seeking behavior. Data in our study indicates that most of our respondents (495, 53.6%) obtained their ED knowledge through the Internet and were less reliant on the doctor (137, 14.9%). Furthermore, lifestyle modification was the most preferred treatment method (654, 70.8%), followed by herbal/traditional treatment (339, 36.7%), but was less on oral medications (277, 30%) and other medical interventions. In Nigeria [20], 39.4% of the respondents were aware of the treatment option for ED, wherein one-fifth (20.4%) had consulted, and 26.5% were able to discuss their sexual challenges with their doctors. However, among Vietnamese married men [21], during the experience of ED, a doctor's advice was also the first line of choice for medical help (55.5%), followed by discussing sexual problems with their wives or partners (55.1%), and 23.1% would seek advice from their friends.

Study limitations are reliance on self-reported data via a questionnaire that may introduce recall and social desirability biases, potentially underreporting sensitive information and overrepresenting socially favorable responses. The use of an online survey could introduce selection bias, possibly excluding individuals without Internet access or technological familiarity. Furthermore, the study's sample may not fully represent the Saudi male population due to convenience sampling and the exclusion of certain demographic groups, diminishing the generalizability of findings. 

## Conclusions

In this study, a significant number of adult Saudi men were found to have ED, particularly those who were older, had higher body mass indices, and suffered from chronic conditions like hypertension and diabetes. Independent risk factors identified included marital status, employment status, and a history of perineum surgery. The study also revealed a general reluctance among men to discuss ED with healthcare providers, preferring instead to seek information online. Most men favored lifestyle modifications over medication or other treatments for ED. There is a clear need for targeted sexual health education and improved community healthcare services focusing on men's sexual health.

## References

[1] (1993). NIH consensus conference. Impotence. NIH consensus development panel on impotence. JAMA.

[2] Kongkanand A (2000). Prevalence of erectile dysfunction in Thailand. Thai erectile dysfunction epidemiological study group. Int J Androl.

[3] Kubin M, Wagner G, Fugl-Meyer AR (2003). Epidemiology of erectile dysfunction. Int J Impot Res.

[4] Bortolotti A, Parazzini F, Colli E, Landoni M (1997). The epidemiology of erectile dysfunction and its risk factors. Int J Androl.

[5] Rodríguez Vela L, Gonzalvo Ibarra A, Pascual Regueiro D, Rioja Sanz LA (2002). Erectile dysfunction (Article in Spanish). Actas Urol Esp.

[6] Selvin E, Burnett AL, Platz EA (2007). Prevalence and risk factors for erectile dysfunction in the US. Am J Med.

[7] El-Sakka AI, Tayeb KA (2003). Erectile dysfunction risk factors in noninsulin dependent diabetic Saudi patients. J Urol.

[8] Rosen RC, Cappelleri JC, Smith MD, Lipsky J, Peña BM (1999). Development and evaluation of an abridged, 5-item version of the International Index of Erectile Function (IIEF-5) as a diagnostic tool for erectile dysfunction. Int J Impot Res.

[9] Zhang X, Yang B, Li N, Li H (2017). Prevalence and risk factors for erectile dysfunction in Chinese adult males. J Sex Med.

[10] Ponholzer A, Temml C, Mock K, Marszalek M, Obermayr R, Madersbacher S (2005). Prevalence and risk factors for erectile dysfunction in 2869 men using a validated questionnaire. Eur Urol.

[11] Miller KE (2000). Does glycemic control correlate with erectile dysfunction. Am Fam Physician.

[12] Ahmed AM (2002). History of diabetes mellitus. Saudi Med J.

[13] Cefalu WT, Weir GC (2003). New technologies in diabetes care. Gale Academic Onefile.

[14] Herrick JB (1901). The diagnosis of diabetes mellitus. J Am Med Assoc.

[15] Khatib FA, Jarrah NS, Shegem NS, Bateiha AM, Abu-Ali RM, Ajlouni KM (2006). Sexual dysfunction among Jordanian men with diabetes. Saudi Med J.

[16] Ahn TY, Park JK, Lee SW (2007). Prevalence and risk factors for erectile dysfunction in Korean men: results of an epidemiological study. J Sex Med.

[17] Al Helali NS, Abolfotouh MA, Ghanem HM (2001). Pattern of erectile dysfunction in Jeddah city. Saudi Med J.

[18] Nasser J, Habib F, Al Saad A, Al Hashmi DA, Abdulla A, Al Tajer LA (20151). Prevalence of risk factors of erectile dysfunction among men with diabetes. Bahrain Med Bull.

[19] Oyelade BO, Jemilohun AC, Aderibigbe SA (2016). Prevalence of erectile dysfunction and possible risk factors among men of South-Western Nigeria: a population based study. Pan Afr Med J.

[20] Abu S, Atim T, Ripiye NR (2019). Prevalence of erectile dysfunction and awareness of its treatment in Abuja, Nigeria. Int J Trop Dis Health.

[21] Van Vo T, Hoang HD, Thanh Nguyen NP (2017). Prevalence and associated factors of erectile dysfunction among married men in Vietnam. Front Public Health.

[22] Li JZ, Maguire TA, Zou KH, Lee LJ, Donde SS, Taylor DG (2022). Prevalence, comorbidities, and risk factors of erectile dysfunction: results from a prospective real-world study in the United Kingdom. Int J Clin Pract.

